# Supplementary material for: Anti-Citrullinated Protein Antibody Titers Are Independently Modulated by Both Disease Activity and Conventional or Biologic Anti-Rheumatic Drugs
Source: Diagnostics (Basel). 2022 Jul 21;12(7):1773. doi: 10.3390/diagnostics12071773 (PMC9319415; doi:10.3390/diagnostics12071773)
Supplement: Supplementary file 1 [file diagnostics-12-01773-s001.zip › diagnostics-1781435-supplementary.pdf]

Supplementary material

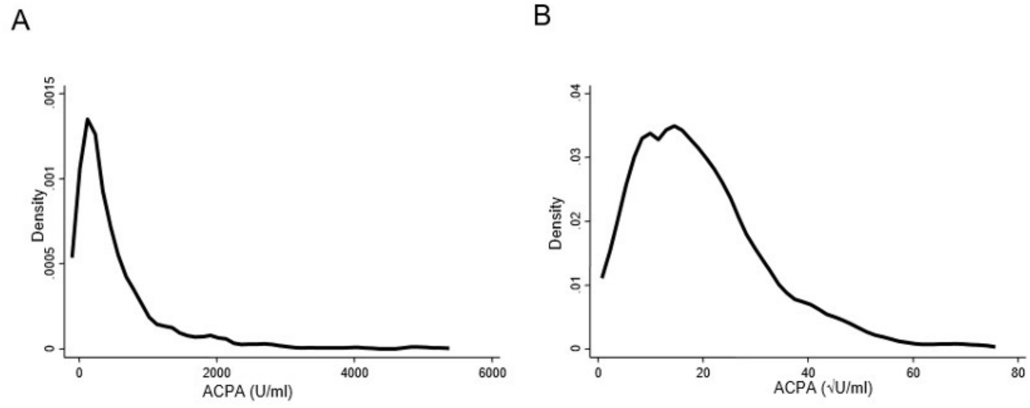

Figure S1. Distribution of anti-CCP2 titers. (**A**) Raw data. (**B**) Square root transformation of values.
